# Urine metabolic profile in rats with arterial hypertension of different genesis

**DOI:** 10.18699/vjgb-24-34

**Published:** 2024-06

**Authors:** A.A. Sorokoumova, A.A. Seryapina, Yu.K. Polityko, L.V. Yanshole, Yu.P. Tsentalovich, М.А. Gilinsky, А.L. Markel

**Affiliations:** Institute of Cytology and Genetics of the Siberian Branch of the Russian Academy of Sciences, Novosibirsk, Russia; Institute of Cytology and Genetics of the Siberian Branch of the Russian Academy of Sciences, Novosibirsk, Russia; Institute of Cytology and Genetics of the Siberian Branch of the Russian Academy of Sciences, Novosibirsk, Russia Scientific Research Institute of Neurosciences and Medicine, Novosibirsk, Russia; International Tomography Center of the Siberian Branch of the Russian Academy of Sciences, Novosibirsk, Russia; International Tomography Center of the Siberian Branch of the Russian Academy of Sciences, Novosibirsk, Russia; Scientific Research Institute of Neurosciences and Medicine, Novosibirsk, Russia; Institute of Cytology and Genetics of the Siberian Branch of the Russian Academy of Sciences, Novosibirsk, Russia Novosibirsk State University, Novosibirsk, Russia

**Keywords:** arterial hypertension, ISIAH rats, L-NAME, DOCA-salt hypertension, urine metabolomic markers, артериальная гипертония, крысы НИСАГ (ISIAH), L-NAME;, DOCA-солевая гипертония, мломные маркеры мочи

## Abstract

The diversity of pathogenetic mechanisms underlying arterial hypertension leads to the necessity to devise a personalized approach to the diagnosis and treatment of the disease. Metabolomics is one of the promising methods for personalized medicine, as it provides a comprehensive understanding of the physiological processes occurring in the body. The metabolome is a set of low-molecular substances available for detection in a sample and representing intermediate and final products of cell metabolism. Changes in the content and ratio of metabolites in the sample mark the corresponding pathogenetic mechanisms by highlighting them, which is especially important for such a multifactorial disease as arterial hypertension. To identify metabolomic markers for hypertensive conditions of different origins, three forms of arterial hypertension (AH) were studied: rats with hereditary AH (ISIAH rat strain); rats with AH induced by L-NAME administration (a model of endothelial dysfunction with impaired NO production); rats with AH caused by the administration of deoxycorticosterone in combination with salt loading (hormone-dependent form – DOCA-salt AH). WAG rats were used as normotensive controls. 24-hour urine samples were collected from all animals and analyzed by quantitative NMR spectroscopy for metabolic profiling. Then, potential metabolomic markers for the studied forms of hypertensive conditions were identified using multivariate statistics. Analysis of the data obtained showed that hereditary stress-induced arterial hypertension in ISIAH rats was characterized by a decrease in the following urine metabolites: nicotinamide and 1-methylnicotinamide (markers of inflammatory processes), N- acetylglutamate (nitric oxide cycle), isobutyrate and methyl acetoacetate (gut microbiota). Pharmacologically induced forms of hypertension (the L-NAME and DOCA+NaCl groups) do not share metabolomic markers with hereditary AH. They are differentiated by N,N-dimethylglycine (both groups), choline (the L-NAME group) and 1-methylnicotinamide (the group of rats with DOCA-salt hypertension).

## Abstract (ru)

Многообразие патогенетических механизмов, лежащих в основе артериальной гипертонии, приводит к необходимости разработки персонализированного подхода к диагностике и терапии заболевания. Одним из перспективных методов для персонализированной медицины является метаболомика, которая позволяет получить комплексное представление о физиологических процессах, происходящих в организме. Метаболом – это совокупность низкомолекулярных веществ, определяемых в образце и являющихся промежуточными и конечными продуктами метаболизма клеток. Изменения в содержании и соотношении метаболитов в исследуемом образце маркируют соответствующие патогенетические механизмы, выделяя их, что особенно важно для такого мультифакторного заболевания, как артериальная гипертония. Для идентификации метаболомных маркеров гипертензивных состояний разного генеза были исследованы три разные формы артериальной гипертонии (АГ): крысы c наследственной АГ (линия крыс НИСАГ/ISIAH); крысы с АГ, индуцированной введением L-NAME (модель эндотелиальной дисфункции с нарушением продукции NO); крысы с АГ, вызванной введением дезоксикортикостерона в сочетании с солевой нагрузкой (гормон-зависимая форма – DOCA-солевая АГ). В качестве нормотензивного контроля были использованы крысы линии WAG. У всех животных собрали образцы суточной мочи, метаболомный профиль которой проанализировали методом количественной ЯМР-спектроскопии. Затем с помощью методов многомерной статистики выявили потенциальные метаболомные маркеры исследуемых форм гипертензивных состояний. Анализ полученных данных показал, что для наследственной стресс-индуцированной артериальной гипертонии у крыс линии НИСАГ характерно снижение содержания следующих метаболитов в моче: никотинамида и 1-метилникотинамида (маркеры воспалительных процессов), N-ацетилглутамата (цикл оксида азота), изобутирата и метилацетоацетата (микробиота кишечника). Фармакологически индуцированные формы АГ (группы L-NAME и DOCA+NaCl) не имеют общих с наследственной АГ метаболомных маркеров. Их отличают один общий маркер, N,N-диметилглицин, и два специфических – холин (для группы L-NAME) и 1-метилникотинамид (для группы крыс с DOCA-солевой артериальной гипертонией).

## Introduction

Arterial hypertension (AH) is a complex multifactorial disease, simultaneously affecting various systems of the body. Pathogenetic diversity of AH and interaction between different underlying mechanisms determine the necessity to consider a lot of factors when developing methods of prevention and treatment. At the moment, multi-stage protocols for the treatment of hypertension have been devised and applied, taking into account lifestyle, stage of the disease, concomitant pathologies, etc. (Carey et al., 2022). However, the tasks of personalized medicine are still relevant, including use of integrated approaches in diagnostics for identifying the distinctive “set” of mechanisms involved in the development of hypertension for a particular patient, and, accordingly, prescribing individual treatment. The so-called “omics” technologies are well suited for this purpose, as they provide a kind of “snapshot” of the organism and its systems for further analysis (at the level of genome, transcriptome, proteome, metabolome, etc.).

To study the metabolic pathways involved in the pathogenesis of various hypertensive conditions, we used three experimental models of hypertension. The first model was the ISIAH rat strain (Inherited Stress-Induced Arterial Hypertension), obtained from an outbred population of Wistar rats through long-term selection for increased blood pressure (BP) under psycho-emotional stress (Markel, 1992). This model reproduces primary (essential) human hypertension quite accurately. The second model – AH caused by dysfunction of the vascular endothelium – was induced pharmacologically by the administration of L-NAME (an inhibitor of NO synthesis) (Biancardi et al., 2007). Endothelial dysfunction associated with impaired nitric oxide synthesis is also one of the common mechanisms of BP rising. The third model of AH was also induced pharmacologically by the administration of a synthetic mineralocorticoid – DOCA (deoxycorticosterone acetate) along with additional salt loading (Basting, Lazartigues, 2017). The combination of elevated mineralocorticoid levels and salt loading is another possible cause of hypertension in humans (Gupta, 2011)

When developing methods to identify biochemical markers for different forms of hypertension, attention should be paid to the availability and non-invasiveness of the proposed technologies. One of the most accessible methods is the analysis of urine samples. Metabolomic studies of urine are currently of interest to researchers, and methods for analyzing and interpreting such data are actively discussed (Zhang et al., 2012; Bouatra et al., 2013).

The purpose of our study is to evaluate the metabolomic profile of 24-hour urine in rats representing three different forms of hypertensive conditions, in comparison with normotensive controls.

## Materials and methods

Experimental animals. We studied 3–4-month-old male rats of the ISIAH strain with a hereditary form of hypertension, together with two groups of rats with pharmacologically induced forms of AH: a group of rats treated with a NO synthesis blocker – L-NAME, and a group of rats with hormonedependent DOCA+NaCl hypertension. WAG rats were used as a normotensive control.

To model NO-deficient hypertension with endothelial dysfunction, WAG rats were orally administered with a solution of endothelial NO synthase inhibitor (L-NAME, Nω-nitrol- arginine methyl ester; Sigma Aldrich, USA) at a dose of 30 mg/kg of body weight for two weeks (Fürstenau et al., 2008). To obtain hormone-dependent DOCA-salt hypertension, WAG rats were subcutaneously injected with DOCA (deoxycorticosterone acetate; Sigma Aldrich, USA) at a dose of 25 mg/kg of body weight once every 4 days with a constant salt loading – 1 % NaCl solution in drinking water – for three weeks (Chan et al., 2006). As a result, four experimental groups of animals were formed, three with hypertension and one normotensive, 10 males in each.

All animals were kept under standard conditions at the vivarium of the Institute of Cytology and Genetics SB RAS (air temperature 22–24 °C, light:dark cycle 12:12 hours), receiving standard chow (Chara, Russia) and free access to drinking water. All procedures with experimental animals complied with the ethical standards approved by the legal acts of the Russian Federation, the principles of the Basel Declaration and the recommendations of the Inter-Institutional Commission on Biological Ethics at the Institute of Cytology and Genetics SB RAS (protocol No. 127 of 09/08/2022).

Blood pressure (BP) monitoring was performed twice a week throughout the experiment on a device for non-invasive BP measurement (BIOPAC, USA) using the tail-cuff method with preliminary adaptation of the animals to this procedure for 3–4 days. Also, simultaneously with BP measurements, the rats were weighed regularly

Collection of 24-hour urine samples. Animals were placed in individual rodent metabolic cages (Techniplast, Italy), where they adapted to new conditions for 3 days. Over the next 3 days, each day at the same time, urine samples were collected and the volume of water drunk was recorded. The collected urine was stored at –70 °C. Further analysis of the samples obtained was carried out at the Center for Shared Use “Mass Spectrometric Research” of the International Tomography Center SB RAS, in the Laboratory of Proteomics and Metabolomics

Extraction of metabolites from urine samples. To obtain a non-protein extract of rat urine metabolites, the following sample preparation protocol was used: the optimal ratio of urine volumes to extracting solution was urine/methanol ratio = 1/4. 400 μl of cold methanol (–20 °C) was added to 100 μl of urine. The samples were mixed in a vortex centrifuge and placed on a shaker for 15 minutes at 1,300 rpm, then centrifuged at 12,000 rpm at 4 °C for 30 minutes, followed by the collection of supernatant. The supernatant was dried on a vacuum evaporator and stored at –70 °C. Lyophilized extracts were diluted in 600 μl of deuterated phosphate buffer (50 mM, pH 7.4) supplemented with internal standard DSS (sodium 3-(trimethylsilyl)propane-1-sulfonate, 20 μM).

NMR spectra were recorded on an AVANCE III HD 700 MHz NMR spectrometer (Bruker BioSpin, Germany), equipped with an Ascend cryomagnet with a field of 16.44 Tesla and a TXI 1H-13C/15N/D ZGR 5 mm probe. Detection parameters corresponded to those described previously (Zelentsova et al., 2020). MestReNova v 12.0 program was used to process the spectra and integrate the signals. Metabolites were identified using the Human Metabolome Database (https:// hmdb.ca/) and our own data on the metabolic profiling of human and animal biological fluids (Tsentalovich et al., 2020; Fomenko et al., 2022).

Statistical data processing was performed using Statistica 12 software package (StatSoft, Inc., 2014) and Metabo Analyst 5.0 web platform (https://www.metaboanalyst.ca/); multivariate analysis (principal component method) and nonparametric methods (Mann–Whitney U test with Bonferroni correction for multiple comparisons) were applied.

## Results

**Fig. 1.**
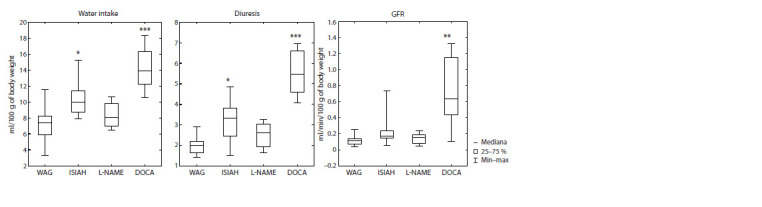
Intergroup differences in daily water intake, daily diuresis and glomerular filtration rate (GFR) in ISIAH, L-NAME, DOCA rats relative to control WAG rats. Mann–Whitney test, * p < 0.05, ** p <0.01, *** p < 0.001.

A comparative analysis of physiological parameters of the studied animals showed that experimental groups of rats did not have significant differences in body weight: WAG – 326.1 ± 12.87 g (BP – 135.9 ± 1.21 mmHg), ISIAH – 325.9 ± 6.44 g (BP – 205.9 ± 2.12 mmHg), L- NAME – 326.9 ± 4.71 g (BP – 192.0 ± 2.96 mmHg), DOCA – 328.2 ± 6.18 g (BP – 184.2 ± 1.19 mmHg). However, daily water intake, daily diuresis and glomerular filtration rate were significantly increased in rats of the DOCA group compared to the control (Fig. 1). Increased diuresis and water intake were also observed in ISIAH rats. Based on these intergroup differences, for metabolomic analysis of daily urine, the concentrations of metabolites obtained by NMR spectroscopy (nmol/ml) were recalculated into daily urine excretion of metabolites (nmol/day), considering the diuresis level in each rat on the day of sample collection.

**Fig. 2.**
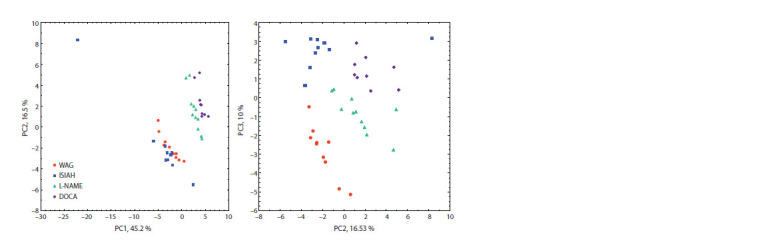
Distribution of groups of normotensive (WAG) and hypertensive (ISIAH, L-NAME, DOCA) rats in the coordinates of the principal component axes (PC1, PC2, PC3).

Multivariate analysis of metabolomic data using the principal component method revealed three main axes (PC1, PC2, PC3), respectively responsible for 45.2, 16.5 and 10.0 % of the total variation in the content of the studied metabolites in 24- hour urine samples. The distribution of experimental groups in the coordinates of the principal components is shown in Fig. 2. A distinct separation of the experimental groups along the axis of the first principal component (responsible for 45.2 % of the variation in the studied parameters) is observed: normotensive WAG rats and ISIAH rats with hereditary hypertension are virtually combined into one group. Rats with pharmacological forms of hypertension – L-NAME and DOCA – form another separate group together. From this we may conclude that “natural” genetic hypertension is in sharp contrast to the two pharmacologically induced forms of AH, so urine metabolomic markers correspond not so much to elevated BP levels as to the pathogenesis of different forms of AH.

A relatively small percentage of variability (16.5 %) in metabolomic parameters is described by the second principal component. The projection of the experimental groups onto the second component does not make it possible to separate the compared groups in accordance to any set of metabolomic markers. At the same time, the residual variability of parameters (10 %) for the third principal component, when considered, showed the possibility of a distinct separation of normotensive rats (WAG) and rats with hereditary arterial hypertension (ISIAH). Thus, metabolomic markers that correlate with the third principal component can serve as diagnostic indicators of hereditary stress-dependent forms of hypertension

**Fig. 3.**
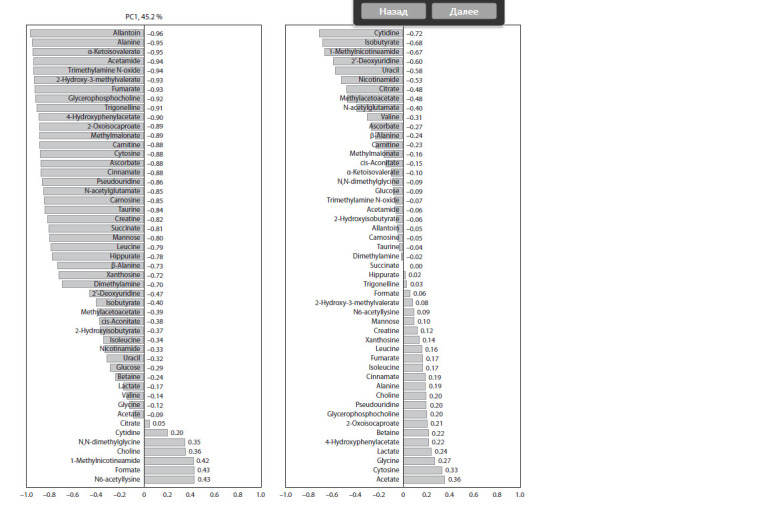
PCA loadings of urine metabolite levels (PC1, PC3).

Fig. 3 shows the loadings of metabolites along the axes of the first and third principal components. For the first principal component, most of the parameters correlate with the combined group – normotensive WAG rats + hypertensive ISIAH rats, – while choline, N,N-dimethylglycine, N6-acetyllysine, 1-methylnicotinamide and formate correlate with pharmacologically induced forms of AH. Thus, the metabolic profile of hereditary AH (at least in the early stages of its development) is closer to normal than to those of pharmacologically induced models of AH. Markers that distinguish ISIAH rats from normotensive controls (WAG rats) may be divided into those that correlate positively and those that correlate negatively with the hereditary form of hypertension, based on their loadings along the axis of the third principal component. Positively correlated compounds include acetate, cytosine, glycine, lactate; negatively correlated are cytidine, isobutyrate, 1-methylnicotinamide, 2′-deoxyuridine, uracil, nicotinamide, citrate, methyl acetoacetate, N-acetylglutamate

In addition to assessing the metabolite loadings along the axes of the principal components, an analysis of intergroup differences in the content of metabolites in the daily urine of the studied animals was also performed (see the Table). Thus, a list of 12 urine metabolites was formed, the levels of which differed from the controls in animals with various forms of AH, also contributing the most to the separation of experimental groups in the coordinates of the principal component axes.

**Table 1.**
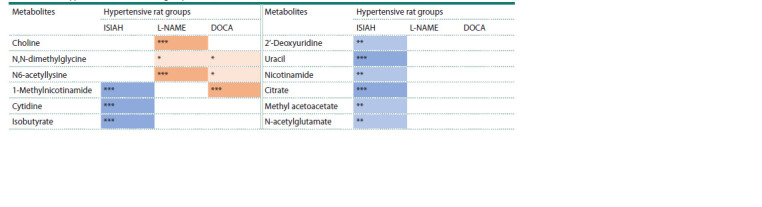
Significant differences in the content of individual metabolites in the urine of hypertensive rats of three groups (ISIAH, L-NAME, DOCA) from the controls Notе. Color indicates an increase (orange) or a decrease (blue) in metabolite levels when compared to control WAG rats. Mann–Whitney test, * p < 0.05, ** p < 0.01, *** p < 0.001.

**Fig. 4.**
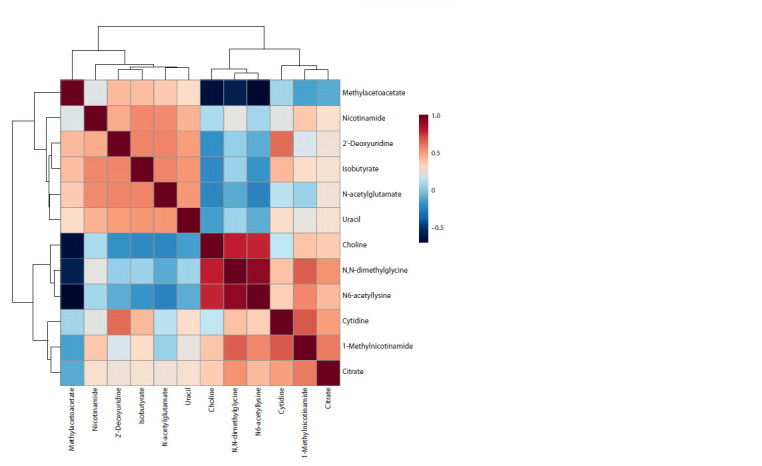
Pearson’s correlation coefficients between the studied parameters (urinary metabolite levels).

To identify possible associations between metabolites that made the greatest contribution to the observed intergroup differences, Pearson’s correlation analysis with partial clustering was performed (Fig. 4). The highest correlation coefficients (r > 0.7) were observed between choline, N,N-dimethylglycine and N6-acetyllysine (the correlation was positive); these three metabolites also correlated negatively with methyl acetoacetate (r < –0.5). Citrate and 1-methylnicotinamide positively correlated with each other, as well as with N,N-dimethylglycine, N6-acetyllysine and cytidine (r > 0.5). Nicotinamide, 2′-deoxyuridine, isobutyrate, N-acetylglutamate and uracil were also positively correlated with each other (r > 0.5). Also, 2′-deoxyuridine positively correlated with cytidine with a correlation coefficient of r = 0.64

Thus, certain associations between urine metabolites were found, which may provide markers for three hypertensive conditions that are different in their genesis.

## Discussion

Choline and homocysteine metabolism

Choline participates in lipid metabolism, in the formation of cell membranes, and in the synthesis of the neurotransmitter acetylcholine (Zeisel, 2000). Choline oxidase and betaine aldehyde dehydrogenase oxidize choline to betaine. Betaine, in its turn, is a methyl group donor for betaine homocysteine methyltransferase that is involved in the remethylation of homocysteine to methionine resulting in the production of N,N-dimethylglycine (an alternative pathway for homocysteine utilization in the folate cycle). Normally, homocysteine should not accumulate in the body; its blood level elevation increases the risk of neurodegenerative and cardiovascular diseases (Wald et al., 2002). An increase in the homocysteine blood concentration causes damage of endothelium, activation of platelet aggregation, and formation of atherosclerotic plaques (Paré et al., 2009; Ganguly, Alam, 2015). Elevated blood levels of N,N-dimethylglycine correlate with increased homocysteine in patients with chronic renal failure (McGregor et al., 2001). Since L-NAME administration inhibits nitric oxide synthesis and leads to endothelial dysfunction, observed simultaneous increase in the levels of choline and N,N-dimethylglycine in the urine of rats in this group is of interest for further research. In the literature, there is evidence of opposite effects of exogenous homocysteine and L-NAME administration – homocysteine increased the expression of NO synthase, which was inhibited by L-NAME (Celotto et al., 2010). At the same time, administration of L-NAME normalized the levels of homocysteine and its metabolites in the blood plasma of rats with cholestasis induced by bile duct ligation (Ebrahimkhani et al., 2005).

N6-acetyllysine is increased in urine of L-NAME and DOCA rats. At this moment, the biological role of this compound has not been determined; however, there is some evidence of its association with the complications of type 1 and type 2 diabetes (Niewczas et al., 2017; Xu et al., 2023). Nevertheless, the lack of description of any pathogenetic mechanisms do not allow us to consider N6-acetyllysine as a potential biomarker of hypertension. High correlation coefficients were observed between N6-acetyllysine, choline and N,N-dimethylglycine. Association of choline and N,N-dimethylglycine with homocysteine and regulation of NO syn-thase was described above – perhaps the role of these compounds in the development of hypertension caused by endothelial dysfunction will be clarified in the future.

Nicotinamide metabolism

Nicotinamide is converted to 1-methylnicotinamide by the liver enzyme nicotinamide-N-methyltransferase. Nicotinamide- N-methyltransferase also promotes remethylation of homocysteine to S-adenosylmethionine (another pathway for homocysteine utilization) (Hong et al., 2018), positively correlates with obesity and insulin resistance (Kannt et al., 2015), and presumably regulates the expression of fructose- 1,6-bisphosphatase involved in the process of gluconeogenesis (Visinoni et al., 2008). Nicotinamide has been shown to prevent cytochrome C release and caspase induction, thereby maintaining mitochondrial membrane potential and exerting a cytoprotective effect on the endothelium of small cerebral vessels (Chong et al., 2002).

There is also evidence that nicotinamide may exhibit anti-inflammatory activity by inhibiting the expression of thromboplastin and CD11b antigen (Ungerstedt et al., 2003). Intravenous administration of 1-methylnicotinamide had an antithrombotic effect by activating the prostacyclin and cyclooxygenase pathways for the inflammation development (Chlopicki et al., 2007). Urine levels of nicotinamide and 1-methylnicotinamide were reduced in ISIAH rats, while in the DOCA-salt group, 1-methylnicotinamide was increased, which may indicate the role of inflammation in both hereditary AH and pharmacologically induced AH pathogenesis.

Pyrimidine metabolism

Serious disorders of pyrimidine metabolism, as a rule, are associated with dysfunction of enzymes, most often of dihydropyrimidine dehydrogenase or dihydropyrimidinase; they occur in early childhood, being systemic in nature and manifesting themselves in mental retardation and seizures (Nyhan, 2005). ISIAH rats do not have such symptoms, although there is a decrease in the levels of cytidine, 2′-deoxyuridine and uracil in their urine compared to normotensive controls. Interpretation of these data is difficult due to only a small number of individual studies: for example, in patients with chronic renal failure, reduced levels of 1-methyladenosine, 1-methylguanosine, N2,N2-dimethylguanosine and N4-acetylcyti- dine renal excretion were found (Niwa et al., 1998). There is also evidence that cyticoline (cytidine-5′-diphosphocholine) synthesized from cytidine and choline may have choline-like effects on membrane metabolism and cholinergic signaling (Yilmaz et al., 2008). However, these results are not sufficient to propose the products of pyrimidine metabolism in urine as markers of a hypertensive state.

Urea and nitric oxide cycle

N-acetylglutamate is an important participant in the urea cycle; it is synthesized in mitochondria from acetyl CoA and glutamate by the enzyme N-acetylglutamate synthase. A deficiency of N-acetylglutamate synthase or N-acetylglutamate itself causes disturbances in the urea cycle and accumulation of free ammonium ions in the blood – hyperammonemia (Tuchman et al., 2008). Increased serum ornithine concentrations have previously been observed in ISIAH rats (Seryapina et al., 2023), which, combined with decreased urinary N-acetylglutamate, suggests that disturbances in nitric oxide synthesis play a significant role in the hypertensive status of ISIAH rats.

Tricarboxylic acid cycle

Citrate is involved in the tricarboxylic acid (TCA) cycle, and a decrease in its urine concentration correlated with the development of hypertension in a study involving volunteers (Chachaj et al., 2020); however, in addition to citrate, the levels of other metabolites participating in the Krebs cycle were also changed: fumarate and trans-aconitate were decreased, methyl malonate was increased. In this study, in ISIAH rats, only citrate urinary level was reduced, so it seems incorrect to claim a serious disturbance of the TCA cycle. Citrate is also known to prevent the crystallization of calcium salts and the formation of kidney stones, therefore, low urine citrate may be linked to the disturbance in the renal mechanism of calcium excretion; besides, there are studies showing association of low citrate excretion with an increased insulin resistance (Cupisti et al., 2007). However, it is difficult to determine the mechanism of citrate decrease in this study, so it seems inappropriate to propose it as a marker of hereditary stressrelated hypertension.

Short-chain fatty acids metabolism

Isobutyrate and methyl acetoacetate are derivatives of the so-called short-chain fatty acids (SCFAs), which are mainly produced by the gut microbiota. A decrease in their production causes intestinal inflammation and dysfunction, and kidney failure, which in turn contributes to increased blood pressure (Kim et al., 2018; Felizardo et al., 2019). It has been shown that short-chain fatty acids can bind to various G protein-coupled receptors. These receptors are located in many tissues and interact with their ligands in different ways (Chen et al., 2020). The effects of SCFAs include modulation of cytokine synthesis, regulation of differentiation and activation of macrophages, neutrophils and T-lymphocytes, and reduction of TNF-α and IL-12 production (Corrêa-Oliveira et al., 2016). In the urine of ISIAH rats, reduced contents of isobutyrate and methyl acetoacetate are observed. Apparently, their hypertensive status is similar in the parameters of the gut microbiota to SHR rats with spontaneous hypertension, in which a reduced number of bacteria producing acetate and butyrate was found (Yang et al., 2015).

## Conclusion

Thus, in ISIAH rats with hereditary stress-dependent hypertension, judging by the characteristics of urine metabolites, disturbances in the nitric oxide cycle (decreased levels of N-acetylglutamate), changes in the function of gut microbiota (decreased isobutyrate and methyl acetoacetate), and the participation of inflammatory processes in formation of hypertensive status (decrease in nicotinamide and 1-methylnicotinamide levels) may be suggested. The data obtained complement our previous study (Seryapina et al., 2023): when comparing serum metabolic profiles of ISIAH and WAG rats, alterations in the nitric oxide cycle were also found (increased ornithine blood level in ISIAH rats), as well as changes in content and ratio of SCFAs (increased isobutyrate and decreased 2-hydroxyisobutyrate), and decreased concentrations of betaine and tryptophan, which have anti-inflammatory properties. Therefore, a decrease in the urinary N-acetylglutamate, isobutyrate, methyl acetoacetate, nicotinamide and 1-methylnicotinamide, in combination with the changes in the serum metabolome listed above, may be considered as a set of potential markers for hereditary stress-dependent hypertension in ISIAH rats.

Pharmacologically induced forms of AH (L-NAME and DOCA+NaCl) are identified by different metabolomic markers (increased urinary levels of choline, N,N-dimethylglycine, 1-methylnicotinamide). These two groups are positioned in the coordinates of the first two principal components in such a way that they actually do not overlap with the groups of control rats and ISIAH rats with hereditary AH. This occurs despite the fact that a common metabolic link is suggested between ISIAH and L-NAME rats, associated with impairment of endothelial function, and, possibly, NO synthesis. The results obtained demonstrate substantial metabolic differences between the “naturally” developing hereditary form of AH and two others caused by external pharmacological influences, which, in fact, made it possible to identify a set of specific metabolomic markers of hereditary, or “primary”, hypertension, distinguishing it from symptomatic, or “secondary” one

## Conflict of interest

The authors declare no conflict of interest.
